# Supplementary figures and images for: The anti-tumor activity of tangeretin in esophageal squamous cell carcinoma by inhibiting GLI2-mediated transcription of GPNMB
Source: PLoS One. 2024 Jun 26;19(6):e0291531. doi: 10.1371/journal.pone.0291531 (PMC11207133; doi:10.1371/journal.pone.0291531)

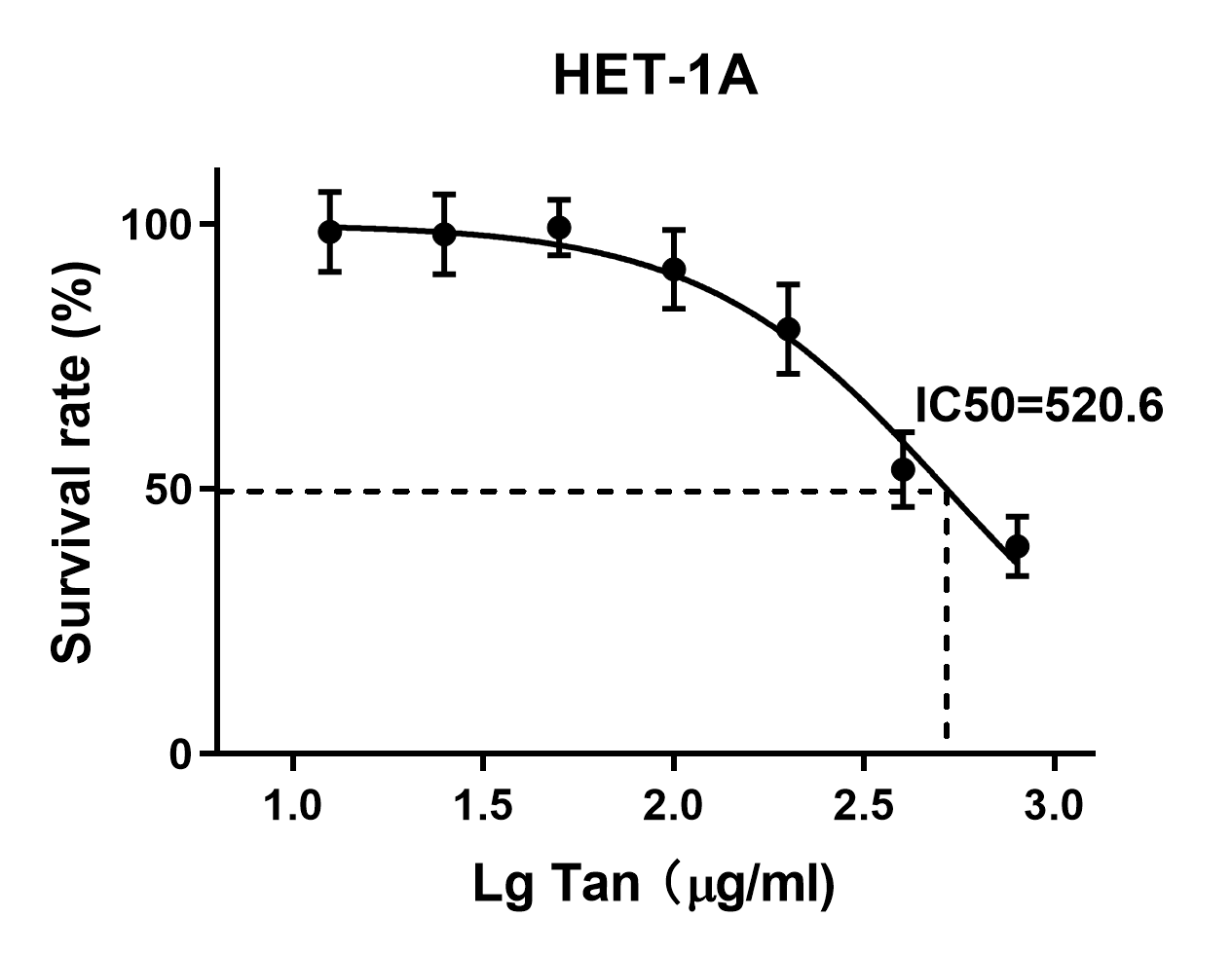

Supplement: S1 Fig — (TIF) [file pone.0291531.s001.tif]

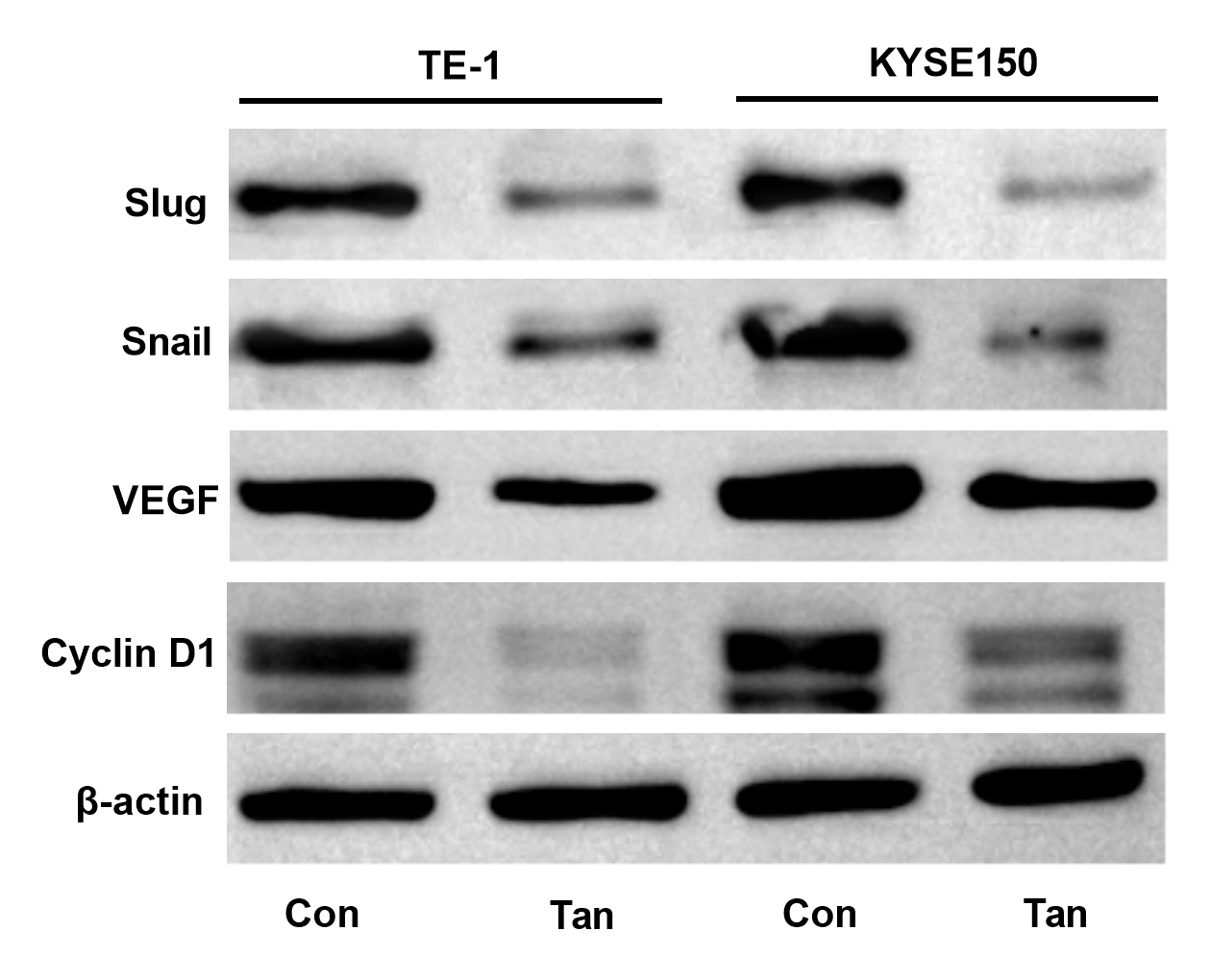

Supplement: S2 Fig — (TIF) [file pone.0291531.s002.tif]
