# Supplementary material for: The anti-tumor activity of tangeretin in esophageal squamous cell carcinoma by inhibiting GLI2-mediated transcription of GPNMB
Source: PLoS One. 2024 Jun 26;19(6):e0291531. doi: 10.1371/journal.pone.0291531 (PMC11207133; doi:10.1371/journal.pone.0291531)

Fig 5C β-actin

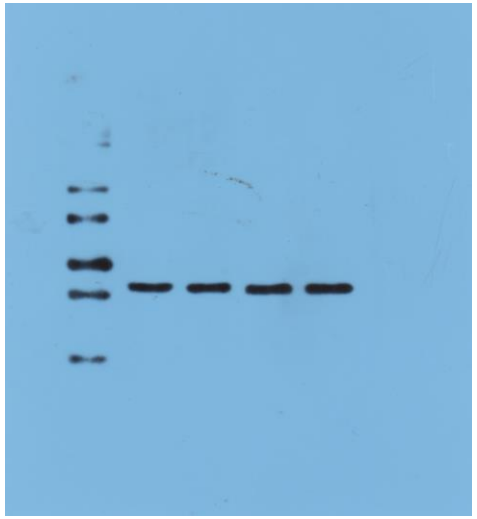

Maker Con Tan Con Tan

TE-1 KYSE150

Fig 5C GLI2

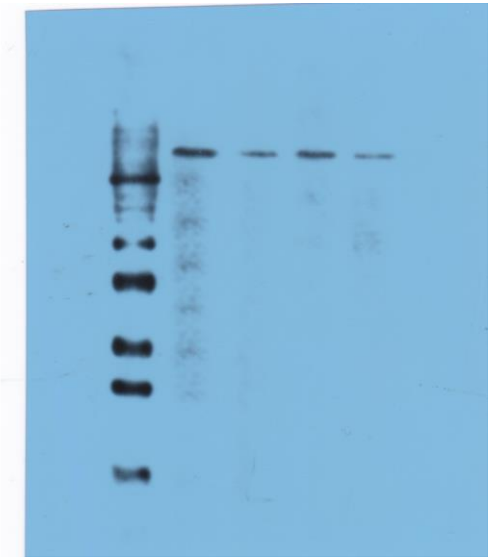

Maker Con Tan Con Tan

TE-1 KYSE150

Fig 6D β-actin

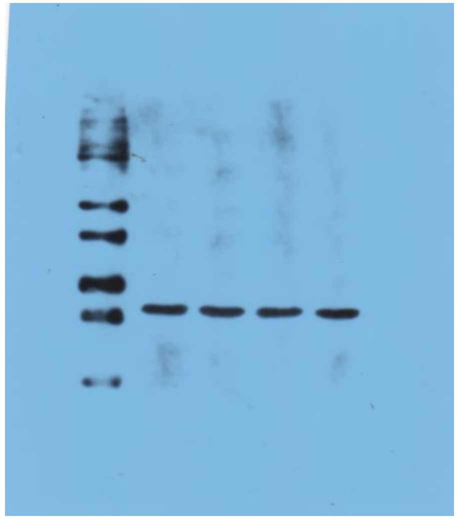

Maker si-NC si-GLI2 si-NC si-GLI2

TE-1 KYSE150

Fig 6D GPNMB

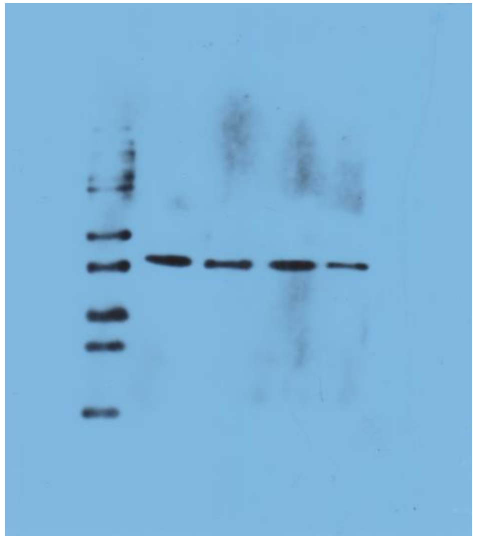

Maker si-NC si-GLI2 si-NC si-GLI2

TE-1 KYSE150

Fig 7E β-actin

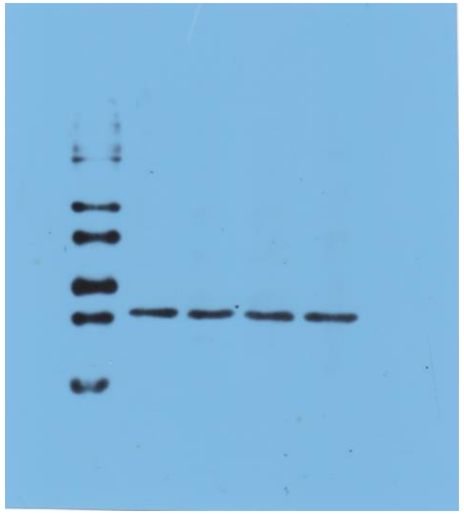

Fig 7E GPNMB

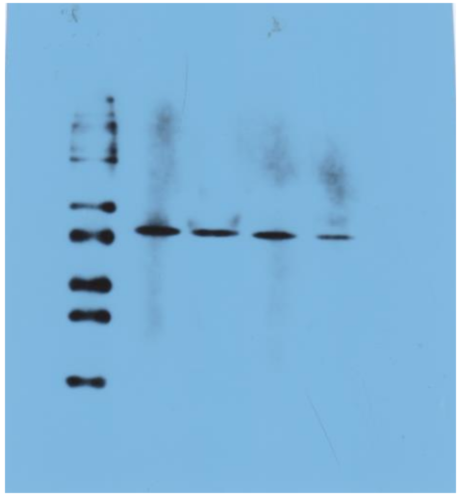

S2 Fig β-actin

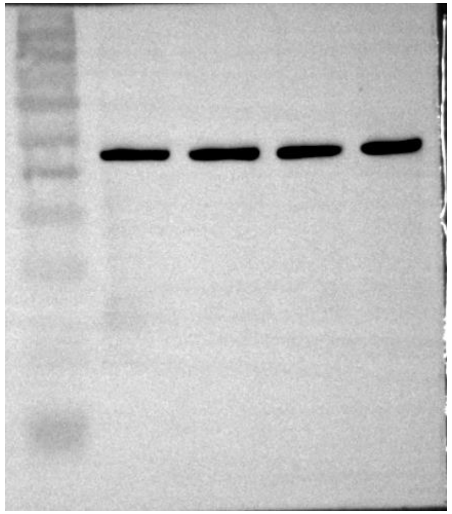

S2 Fig Slug

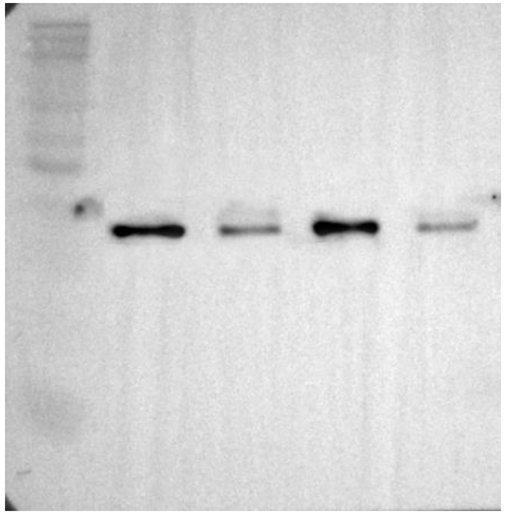

S2 Fig Snail

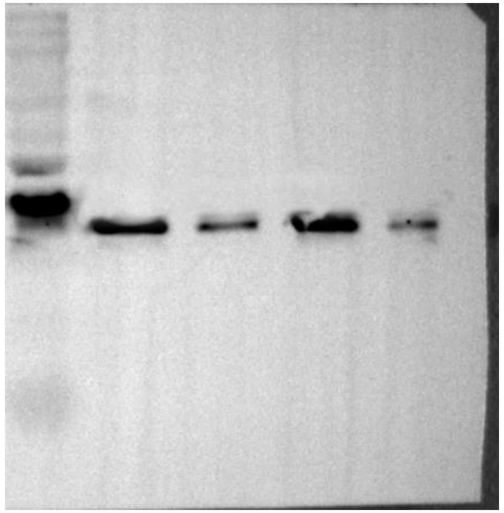

S2 Fig VEGF

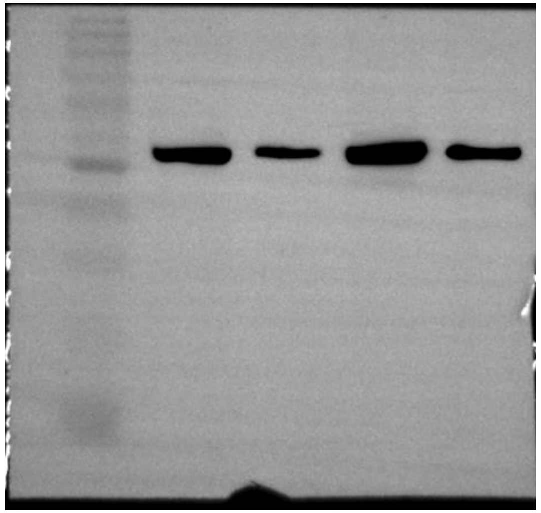

Maker Con Tan Con Tan

TE-1 KYSE150

S2 Fig Cyclin D1

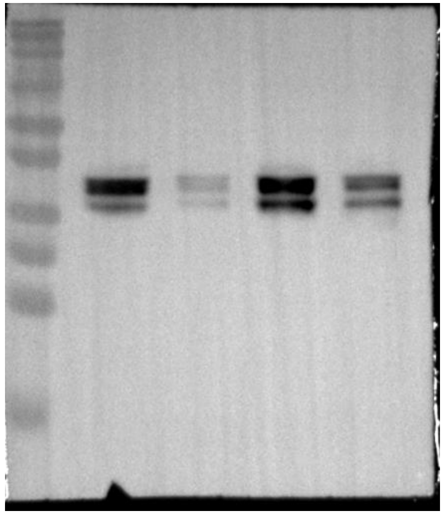

Maker Con Tan Con Tan

TE-1 KYSE150

Fig 1B TE-1

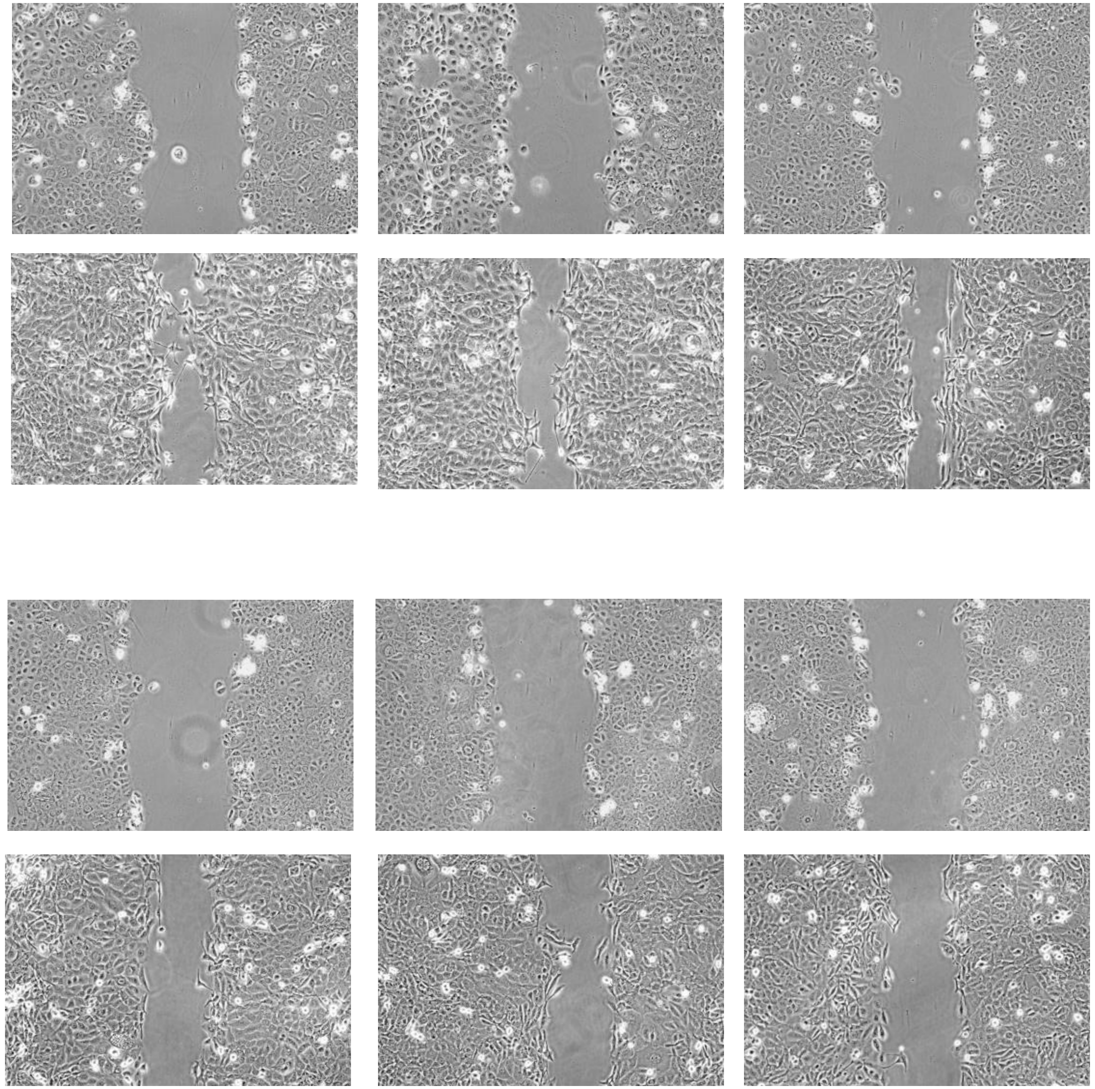

Fig 1B KYSE150

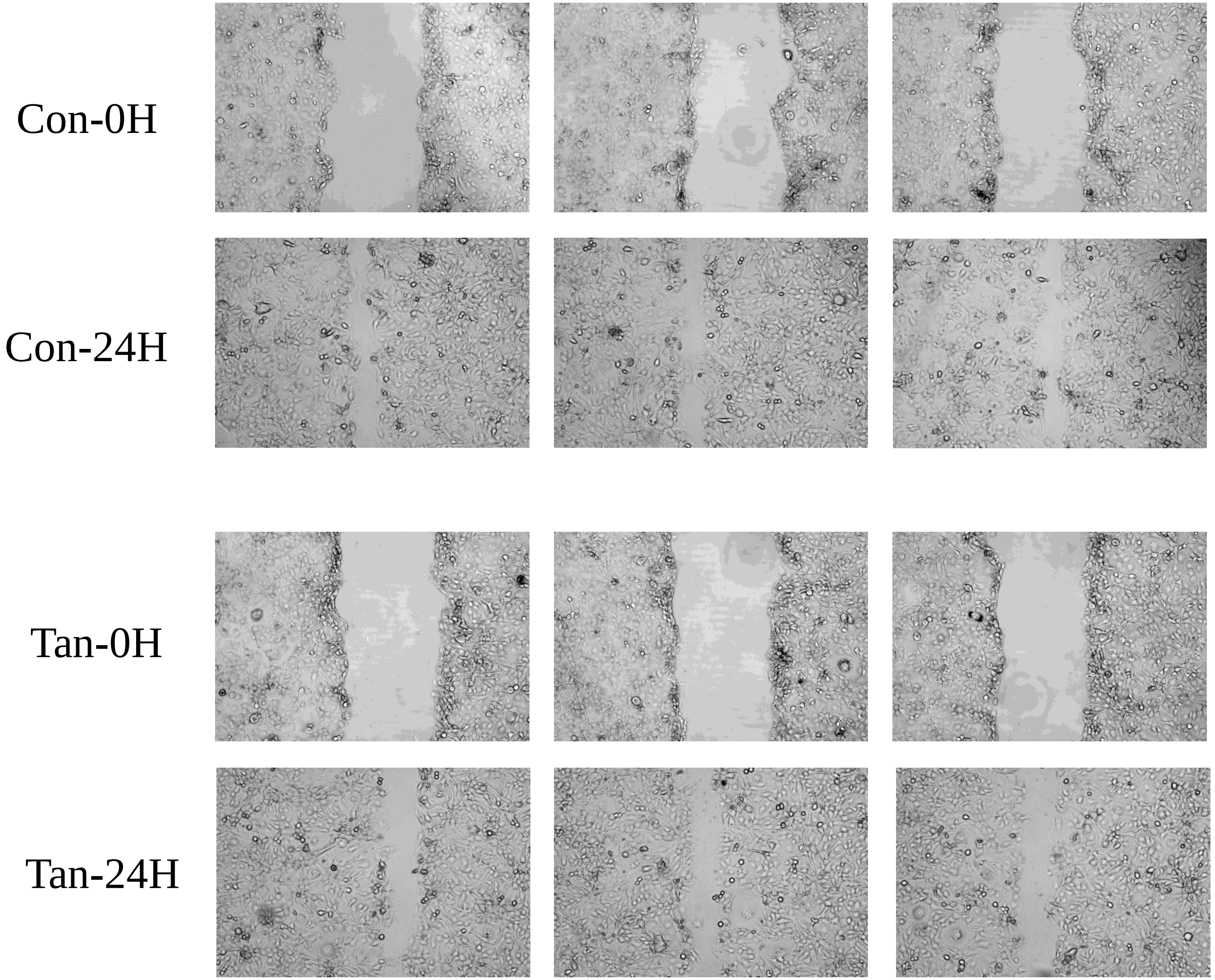

Fig 8C TE-1

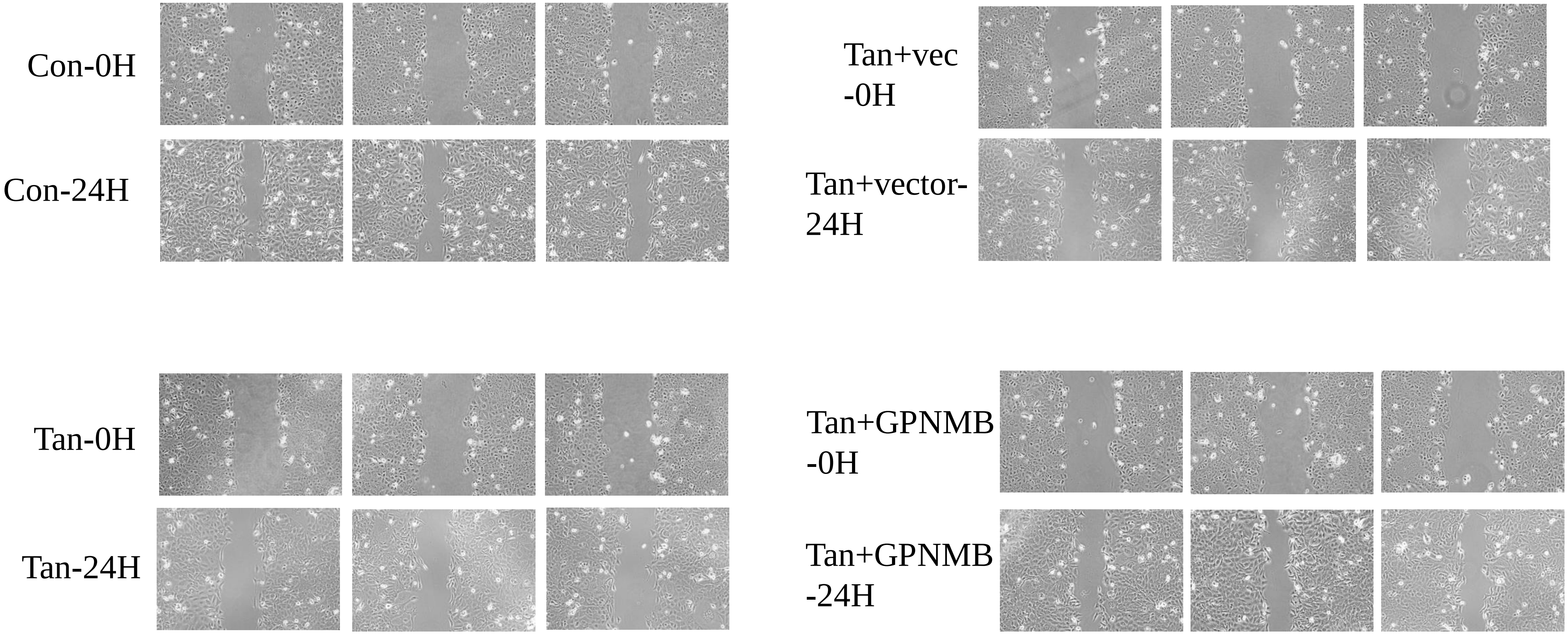
Con-0H
Con-24H
Tan+vec
-0H
Tan+vector-
24H
Tan-0H
Tan-24H
Tan+GPNMB
-0H
Tan+GPNMB
-24H

Fig 8C KYSE150

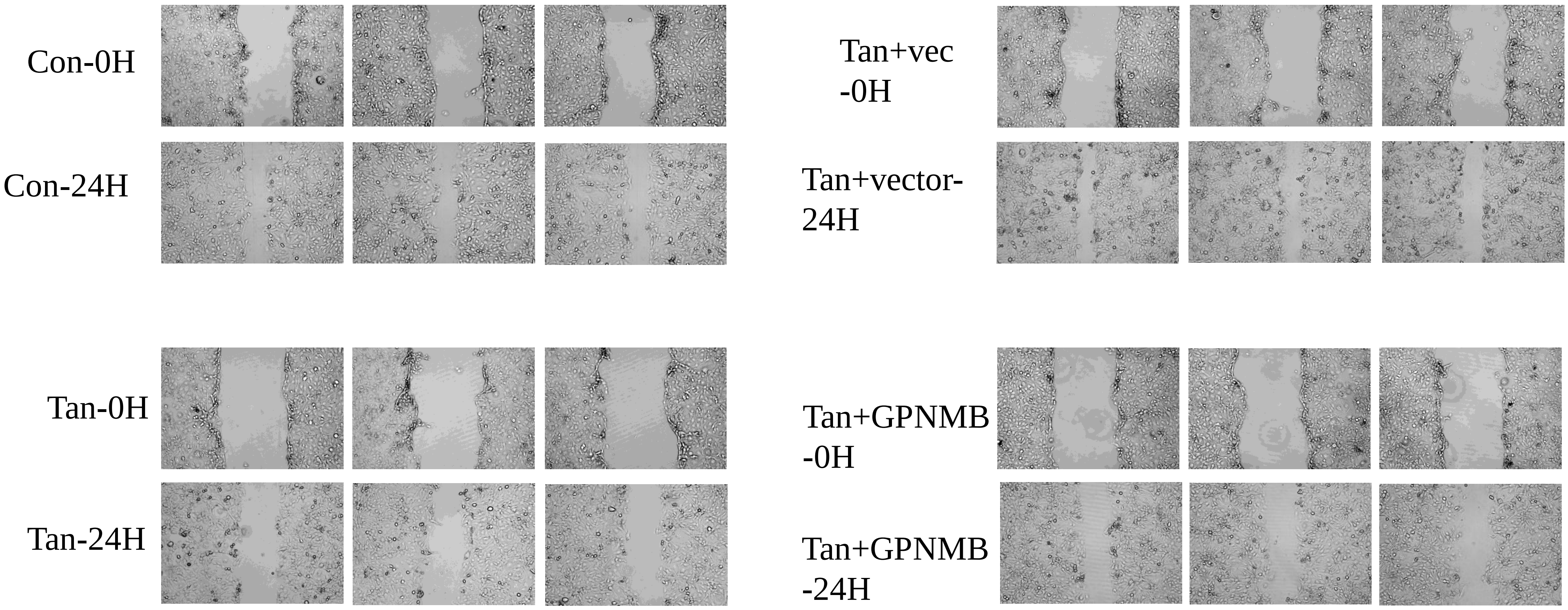

Supplement: S1 Raw images — (PDF) [file pone.0291531.s007.pdf]
