# Supplementary material for: The anti-tumor activity of tangeretin in esophageal squamous cell carcinoma by inhibiting GLI2-mediated transcription of GPNMB
Source: PLoS One. 2024 Jun 26;19(6):e0291531. doi: 10.1371/journal.pone.0291531 (PMC11207133; doi:10.1371/journal.pone.0291531)
Supplement: Attachment — Submitted filename: Response to Reviewers.docx [file pone.0291531.s008.docx]

PONE-D-23-03144

The anti-tumor activity of tangeretin in esophageal squamous cell carcinoma by inhibiting GLI2-mediated transcription of GPNMB

PLOS ONE

Please include the following items when submitting your revised manuscript:

- A rebuttal letter that responds to each point raised by the academic editor and reviewer(s). You should upload this letter as a separate file labeled 'Response to Reviewers'.
- A marked-up copy of your manuscript that highlights changes made to the original version. You should upload this as a separate file labeled 'Revised Manuscript with Track Changes'.
- An unmarked version of your revised paper without tracked changes. You should upload this as a separate file labeled 'Manuscript'.

If you would like to make changes to your financial disclosure, please include your updated statement in your cover letter. Guidelines for resubmitting your figure files are available below the reviewer comments at the end of this letter.

If applicable, we recommend that you deposit your laboratory protocols in protocols.io to enhance the reproducibility of your results. Protocols.io assigns your protocol its own identifier (DOI) so that it can be cited independently in the future. For instructions see: <https://journals.plos.org/plosone/s/submission-guidelines#loc-laboratory-protocols>. Additionally, PLOS ONE offers an option for publishing peer-reviewed Lab Protocol articles, which describe protocols hosted on protocols.io. Read more information on sharing protocols at <https://plos.org/protocols?utm_medium=editorial-email&utm_source=authorletters&utm_campaign=protocols>.

We look forward to receiving your revised manuscript.

Kind regards,

Arunava Roy, Ph.D.
Academic Editor
PLOS ONE

Dear Editor,

We have revised the manuscript according to Reviewers’ comments and journal requirements. We look forward to hearing from you. Thank you very much.

**Journal Requirements:**

When submitting your revision, we need you to address these additional requirements.

1. Please ensure that your manuscript meets PLOS ONE's style requirements, including those for file naming. The PLOS ONE style templates can be found at 
https://journals.plos.org/plosone/s/file?id=wjVg/PLOSOne_formatting_sample_main_body.pdf and 
https://journals.plos.org/plosone/s/file?id=ba62/PLOSOne_formatting_sample_title_authors_affiliations.pdf

Response: We have revised the style and ensured that our manuscript meets PLOS ONE’s style requirements.

2. To comply with PLOS ONE submissions requirements, in your Methods section, please provide additional information regarding the experiments involving animals and ensure you have included details on (1) methods of sacrifice, (2) methods of anesthesia and/or analgesia, and (3) efforts to alleviate suffering.

Response: We have added the details about the above questions in the Revised manuscript.

3. We note that you have indicated that data from this study are available upon request. PLOS only allows data to be available upon request if there are legal or ethical restrictions on sharing data publicly. For more information on unacceptable data access restrictions, please see http://journals.plos.org/plosone/s/data-availability#loc-unacceptable-data-access-restrictions.

In your revised cover letter, please address the following prompts:

a) If there are ethical or legal restrictions on sharing a de-identified data set, please explain them in detail (e.g., data contain potentially sensitive information, data are owned by a third-party organization, etc.) and who has imposed them (e.g., an ethics committee). Please also provide contact information for a data access committee, ethics committee, or other institutional body to which data requests may be sent.

b) If there are no restrictions, please upload the minimal anonymized data set necessary to replicate your study findings as either Supporting Information files or to a stable, public repository and provide us with the relevant URLs, DOIs, or accession numbers. For a list of acceptable repositories, please see http://journals.plos.org/plosone/s/data-availability#loc-recommended-repositories.

We will update your Data Availability statement on your behalf to reflect the information you provide.

Response: We have provided raw images including all western blots and wound-healing assays in the Supporting Information.

4. PLOS ONE now requires that authors provide the original uncropped and unadjusted images underlying all blot or gel results reported in a submission’s figures or Supporting Information files. This policy and the journal’s other requirements for blot/gel reporting and figure preparation are described in detail at https://journals.plos.org/plosone/s/figures#loc-blot-and-gel-reporting-requirements and https://journals.plos.org/plosone/s/figures#loc-preparing-figures-from-image-files. When you submit your revised manuscript, please ensure that your figures adhere fully to these guidelines and provide the original underlying images for all blot or gel data reported in your submission. See the following link for instructions on providing the original image data: https://journals.plos.org/plosone/s/figures#loc-original-images-for-blots-and-gels. 
  
In your cover letter, please note whether your blot/gel image data are in Supporting Information or posted at a public data repository, provide the repository URL if relevant, and provide specific details as to which raw blot/gel images, if any, are not available. Email us at plosone@plos.org if you have any questions.

Response: We have provided all raw images for western blot in the Supporting Information in the Revised manuscript.

5. PLOS requires an ORCID iD for the corresponding author in Editorial Manager on papers submitted after December 6th, 2016. Please ensure that you have an ORCID iD and that it is validated in Editorial Manager. To do this, go to ‘Update my Information’ (in the upper left-hand corner of the main menu), and click on the Fetch/Validate link next to the ORCID field. This will take you to the ORCID site and allow you to create a new iD or authenticate a pre-existing iD in Editorial Manager. Please see the following video for instructions on linking an ORCID iD to your Editorial Manager account: <https://www.youtube.com/watch?v=_xcclfuvtxQ>

Response: We have added the ORCID iD of the corresponding author in the Revised manuscript.

6. Please include captions for your Supporting Information files at the end of your manuscript, and update any in-text citations to match accordingly. Please see our Supporting Information guidelines for more information: <http://journals.plos.org/plosone/s/supporting-information>.

Response: We have added captions for the Supporting Information files at the end of our Revised manuscript.

7. Please review your reference list to ensure that it is complete and correct. If you have cited papers that have been retracted, please include the rationale for doing so in the manuscript text, or remove these references and replace them with relevant current references. Any changes to the reference list should be mentioned in the rebuttal letter that accompanies your revised manuscript. If you need to cite a retracted article, indicate the article’s retracted status in the References list and also include a citation and full reference for the retraction notice.

[Note: HTML markup is below. Please do not edit.]
Response: We have checked the reference list and ensured that no retracted documents were cited in our manuscript. We have also revised the reference style according to the PLOS ONE's style requirements in the Revised manuscript.

**Reviewers' comments:**
Reviewer's Responses to Questions

**Comments to the Author**

1. Is the manuscript technically sound, and do the data support the conclusions?
The manuscript must describe a technically sound piece of scientific research with data that supports the conclusions. Experiments must have been conducted rigorously, with appropriate controls, replication, and sample sizes. The conclusions must be drawn appropriately based on the data presented.

Reviewer #1: Yes

Reviewer #2: Yes

Reviewer #3: Yes

2. Has the statistical analysis been performed appropriately and rigorously?

Reviewer #1: Yes

Reviewer #2: Yes

Reviewer #3: Yes

3. Have the authors made all data underlying the findings in their manuscript fully available?

The [PLOS Data policy](http://www.plosone.org/static/policies.action#sharing) requires authors to make all data underlying the findings described in their manuscript fully available without restriction, with rare exception (please refer to the Data Availability Statement in the manuscript PDF file). The data should be provided as part of the manuscript or its supporting information, or deposited to a public repository. For example, in addition to summary statistics, the data points behind means, medians and variance measures should be available. If there are restrictions on publicly sharing data—e.g. participant privacy or use of data from a third party—those must be specified.

Reviewer #1: Yes

Reviewer #2: Yes

Reviewer #3: Yes

4. Is the manuscript presented in an intelligible fashion and written in standard English?

PLOS ONE does not copyedit accepted manuscripts, so the language in submitted articles must be clear, correct, and unambiguous. Any typographical or grammatical errors should be corrected at revision, so please note any specific errors here.

Reviewer #1: Yes

Reviewer #2: Yes

Reviewer #3: Yes

5. Review Comments to the Author

Please use the space provided to explain your answers to the questions above. You may also include additional comments for the author, including concerns about dual publication, research ethics, or publication ethics. (Please upload your review as an attachment if it exceeds 20,000 characters)

**Reviewer #1:**

1. Provide at least a reference for the given methodology mentioned in materials and methods.

Response: Thank you for your valuable suggestion. We have provided references for the methods in the Revised manuscript.

2. In table 1, check the page format which affected the name EN1

Response: Thank you. We have checked the page format, and revised it.

3. Provide the scale bar for fig.1 & 7 B, arrange an original (un-edited) figure for the fig.1&7 B (Wound-healing assay) in supplementary info.

Response: Thank you for your kind recommendations. We have added the scale bars in Revised Figure 1 and 7 and manuscript. We also provided the original figures for wound-healing assay in the Supporting information.

4. Figure 1 looks packed, it’s better to split into 2 figures for better visualization.

Response: We have split the Figure 1 into 2 figures in the Revised manuscript.

5. In the figure 1E and F, the resolution is poor to understand the differences between treated versus control. Increase resolution and mark the changes with arrow.

Response: Thank you for your valuable suggestion. We have increased resolution in the Revised Figure 2A and 2B. In these assays, Tan reduced the expression of PCNA and MMP9 in ESCC cells, as presented by the reduced mean green fluorescence intensity.
6. Provide the approval info in manuscript, regarding animal study and ethical concerns.

Response: We have provided the approval info in manuscript, regarding animal study and ethical concerns in the Revised manuscript as below.

All animal procedures were conducted in accordance with a protocol approved by Animal Care and Use Committee of Henan Provincial People's Hospital. (page 9, paragraph 2)

7. The figure 2D&E about Immunofluorescence for PCNA and MMP9, the finding are not clear. More resolution and highlight the difference.

Response: According to the comment, we have provided more resolution pictures in the Revised Figure 3D and 3E. In these assays, Tan reduced the expression of PCNA and MMP9 in xenograft tumors, as presented by the reduced PCNA positive cells (red-stained cells) and mean red fluorescence intensity of MMP9, respectively.

8. Unable to read the legends of figure 3 and 5, please increase the resolution and font size.

Response: Thank you for your valuable suggestion. We have increased the solution and font size in the Revised Figure 4 and 6.

**Reviewer #2:**

In this manuscript, Quan Zhang et al., describes the anti-migration and anti-invasion properties of a flavonoid natural product tangeretin in esophageal squamous cell carcinoma (ESCC) cells. In xenograft tumor mouse model, tangeretin reduced the tumor growth and decreased the proliferation markers. Using gene silencing experiments, Chip assays, RNA seq and bioinformatic analysis, the authors have shown that suppression of GLI-2 mediated GPNMB transcription is responsible for the observed anticancer activity. Even though various publications highlight the potential anticancer properties of tangeretin, the results presented in this manuscript is interesting and the studies were well planned and executed. I would recommend this manuscript after addressing the following minor issues.
1. Scale bars should be included in figures or figure legends where fluorescent images were reported.

Response: Thank you for your valuable suggestion. We have added scale bars in Revised figures and figure legends.

2. The author showed that tangeretin suppressed GLI-2 mediated GPNMB transcription in ESCC cells. Through in vitro and in vivo experiments, the authors showed that tangeretin reduced the expression of MMP-9 levels. Is there a shared mechanism behind these two processes, or does GLI2 downregulation result in decreased MMP-9 expression in ESCC? For example, various articles (example. Chin J Cancer Res. 25(6), 637–645) explaining the role of MMP-9 in ESCC and other cancers. Especially, Faião-Flores et al., (Oncogene, 36, 2017, 1849–186) showed that downregulating GLI2 expression leads to reduced MMP-9 expression in melanoma cell lines. In line with these findings, I would recommend the authors to briefly discuss about this relationship in the manuscript.

Response: Thank you for your valuable suggestion. We have discussed the related relationship in the Revised manuscript as below.

Additionally, MMP9 is a crucial regulator implicated in ESCC outcome and prognosis [41], and its expression can be regulated by GLI2 in melanoma cells [42]. A previous document proved the modulation of GPNMB in MMP9 in chronic obstructive pulmonary disease [43]. Our data also showed that Tan can down-regulate MMP9 expression by diminishing GPNMB level in ESCC cells. With these findings, we speculate that Tan in ESCC cells down-regulates MMP9 expression by suppressing GLI2-mediated GPNMB transcription. (discussion section, page 16-17, paragraph 2)

3. Even though tangeretin acts by downregulating GLI2 transcription factor, the molecular target of tangeretin is still unknown. I would encourage the authors to briefly comment about the upstream activators of GLI2 as the possible molecular target of tangeretin.

Response: According to the comment, we have added the discussion about the mechanism by which tangeretin downregulates GLI2 in the Revised manuscript as below.

In addition to the Hedgehog signaling, GLI2 has identified as a direct target of the TGF-β/SMAD pathway [13]. Previous work also uncovered the inhibition of Tan in TGF-β1 expression in human retinal pigment epithelial cells [36]. These conclusions suggest that Tan may reduce GLI2 expression by suppressing the TGF-β/SMAD pathway. Related field research is warranted in future work. (discussion section, page 15-16, paragraph 3)

4. Is there any weight loss noticed with the treatment group compared to the control group in vivo tumor xenograft studies?

Response: In this study, the generated xenograft tumors have little effect on the animal’s health and behavior. Owing to the light xenograft weight, there was no significant difference in body weight between the treatment group and the control group.
5. In the abstract, background section, the following sentence should be rewritten,
“However, the precise action of Tan in the development of esophageal squamous cell carcinoma (ESCC) has remained a mystery”

Response: We have revised the sentence to “However, the precise role of Tan in the development of esophageal squamous cell carcinoma (ESCC) remains unclear” in the Revised manuscript.

6. The manuscript will get benefit from additional English proof reading.

Response: Thank you for your valuable suggestion. According to the comment, we have re-revised the English in the Revised manuscript as below (for instance).

eg. Functional studies in various cancer cell lines have highlighted the tumor-inhibitory potential of Tan [8-10], but its precise action in ESCC pathogenesis remains unclear. (introduction section, page 12, paragraph 2)

eg. A loop graph was drawn for the visualization of the enrichment results. (method section, page 5, paragraph 2)

eg. We identified 587 genes with a significant variation following Tan treatment in TE-1 cells, in which 361 genes were down-regulated and 226 genes were up-regulated (S1 Table), and the cluster heat map of differentially expressed genes was shown in Fig 4A. (result 3 section, page 11, paragraph 2)

eg. Emerging evidence supports the notion that Tan exhibits potent anti-tumor efficacy against numerous human cancers [5,6]. (discussion section, page 14, paragraph 4)

**Reviewer #3:**

Remarks to the Author:
The article “The anti-tumor activity of tangeretin in esophageal squamous cell carcinoma by
inhibiting GLI2-mediated transcription of GPNMB’’ is an interesting study detecting the effect of tangeretin on migration and invasion in esophageal cancer cells by downregulating GLI2-mediated transcription.
However, the existing data are partially supportive of the authors' conclusions. Additional experiments are needed to fully support their findings. Minor revision is needed in recommending this manuscript for publication.

Minor issues:
1. In Fig.1A the author mentioned that the IC50 of tangeretin in TE-1 and KYSE150 is 85.92 and 94.34 μg/ml respectively, but why the author has selected the dose of 20 μg/ml concentration for evaluation of the anti-migratory effect tangeretin in these cells is unclear. Moreover, the IC50 value of tangeretin in normal esophageal cells need to be included in the result. The effect of tangeretin on normal cell growth and proliferation need to be included.

Response: Thank you for your kind recommendations. Based on the use of 1/5-1/2 IC50 of tangeretin in studies studying the effect of tangeretin on cancer cell behaviors, we used 20 μg/ml concentration (1/4-1/5) to assay the effect of tangeratin on ESCC cell migration and invasion in this study. In this study, we found that 20 μg/ml of tangeretin can significantly repress ESCC cell migration and invasion. When the concentration of tangeratin is high, the cell viability and migration will be significantly inhibited, which is not conducive to the study of the mechanism of tangeratin (rescue experiments). According to the comment, we have added the IC50 value of tangeretin in normal esophageal HET-1A cells to show the effect of tangeretin on cell growth in the S1 Fig and Revised manuscript as below.

Intriguingly, Tan (1-100 µg/ml) did not significantly affect the growth of normal esophageal HET-1A cells, and the IC50 value of Tan was 520.6 µg/ml against HET-1A cells (S1 Fig), suggesting the safety of Tan at low doses as an anti-tumor drug. (page 15, paragraph 2)

2. Authors mentioned that Gli2 downregulation by tangeretin causes reduction in cancer cell migration and invasion. Authors only showed the expression of MMP9 and PCNA, but Gli2 itself controls the transcription of EMT (epithelial-mesenchymal transition) specific molecules like Slug, Snail and also controls molecules like VEGF, Cyclin D1, Cyclin D2, Cyclin E. Hence, the effect of tangeretin on the expression these proteins should be determined by western blot analysis.

Response: Thank you for your valuable comments. According to the comment, we have added the discussion in the Revised manuscript as below.

Some important metastasis-related markers, such as EMT specific molecule Snail, angiogenesis-related molecule VEGF, and cell cycle regulator Cyclin D1, have been identified as target genes of GLI2 TF [39,40]. Our data also uncovered the repression of Tan in the expression of Slug, Snail, VEGF, and Cyclin D1 in ESCC cells (S2 Fig), suggesting that Tan may downregulate their expression via GLI2 and thus influences ESCC metastasis. (page 16, paragraph 2)

3. The resolution of Fig 3 (A-D), Fig. 5 A, B are very poor and should be changed with higher resolution figures.

Response: Thank you for your kind recommendations. We have provided higher resolution figures in the Revised Figure 3 and 5.

6. PLOS authors have the option to publish the peer review history of their article ([what does this mean?](https://journals.plos.org/plosone/s/editorial-and-peer-review-process#loc-peer-review-history)). If published, this will include your full peer review and any attached files.

If you choose “no”, your identity will remain anonymous but your review may still be made public.

**Do you want your identity to be public for this peer review?** For information about this choice, including consent withdrawal, please see our [Privacy Policy](https://www.plos.org/privacy-policy).

Reviewer #1: No

Reviewer #2: No

Reviewer #3: No
