# Supplementary material for: The anti-tumor activity of tangeretin in esophageal squamous cell carcinoma by inhibiting GLI2-mediated transcription of GPNMB
Source: PLoS One. 2024 Jun 26;19(6):e0291531. doi: 10.1371/journal.pone.0291531 (PMC11207133; doi:10.1371/journal.pone.0291531)
Supplement: Attachment — Submitted filename: Review comments.docx [file pone.0291531.s009.docx]

Remarks to the Author:

The article “The anti-tumor activity of tangeretin in esophageal squamous cell carcinoma by

inhibiting GLI2-mediated transcription of GPNMB’’ is an interesting study. Minor revision is needed in recommending this manuscript for publication.

Minor issues:

**The Quality of the images should be increased.**

1. In Fig.1A, 1B, 1C, 1D need to be replaced with high resolution images. All the cell images 1B, 1C need scale bars as many of them lack scale bars into it. In fig. 1A and the graphs in Fig. 1B, 1C and 1D the font size should be increased for clear visibility.

2. In Fig.2 A and 2B all the fluorescent images needed scale bars into it. The font size of the graphs in these figure need to be increased.

3. Fig. 3E needs to be changed with high resolution image.

4. Fig. 4A, 4B, 4C font size need to be increased.

5. Fig. 6A, 6B need to be needs to be changed with high resolution images with increased font size.

6. Font size of fig.7B needs to be increased.

7. Fig. 8C, 8D and 8E needs to be changed with high resolution images and scale bars must be added in each cell images.

8. Fig. 9A, 9B scale bars need to be added in each immunofluorescence cell images.
