# Supplementary material for: The anti-tumor activity of tangeretin in esophageal squamous cell carcinoma by inhibiting GLI2-mediated transcription of GPNMB
Source: PLoS One. 2024 Jun 26;19(6):e0291531. doi: 10.1371/journal.pone.0291531 (PMC11207133; doi:10.1371/journal.pone.0291531)
Supplement: Attachment — Submitted filename: Response to Reviewers.docx [file pone.0291531.s010.docx]

PONE-D-23-03144R1

The anti-tumor activity of tangeretin in esophageal squamous cell carcinoma by inhibiting GLI2-mediated transcription of GPNMB

PLOS ONE

Dear Dr. Yao,

Thank you for submitting your manuscript to PLOS ONE. After careful consideration, we feel that it has merit but does not fully meet PLOS ONE’s publication criteria as it currently stands. Therefore, we invite you to submit a revised version of the manuscript that addresses the points raised during the review process.

The original reviewers reviewed the revised manuscript, and though they overall had positive recommendations, two of them suggested minor changes.

Please submit your revised manuscript by Aug 31 2023 11:59PM. If you will need more time than this to complete your revisions, please reply to this message or contact the journal office at [plosone@plos.org](mailto:plosone@plos.org). When you're ready to submit your revision, log on to https://www.editorialmanager.com/pone/ and select the 'Submissions Needing Revision' folder to locate your manuscript file.

Please include the following items when submitting your revised manuscript:

- A rebuttal letter that responds to each point raised by the academic editor and reviewer(s). You should upload this letter as a separate file labeled 'Response to Reviewers'.
- A marked-up copy of your manuscript that highlights changes made to the original version. You should upload this as a separate file labeled 'Revised Manuscript with Track Changes'.
- An unmarked version of your revised paper without tracked changes. You should upload this as a separate file labeled 'Manuscript'.

If you would like to make changes to your financial disclosure, please include your updated statement in your cover letter. Guidelines for resubmitting your figure files are available below the reviewer comments at the end of this letter.

If applicable, we recommend that you deposit your laboratory protocols in protocols.io to enhance the reproducibility of your results. Protocols.io assigns your protocol its own identifier (DOI) so that it can be cited independently in the future. For instructions see: <https://journals.plos.org/plosone/s/submission-guidelines#loc-laboratory-protocols>. Additionally, PLOS ONE offers an option for publishing peer-reviewed Lab Protocol articles, which describe protocols hosted on protocols.io. Read more information on sharing protocols at <https://plos.org/protocols?utm_medium=editorial-email&utm_source=authorletters&utm_campaign=protocols>.

We look forward to receiving your revised manuscript.

Kind regards,

Arunava Roy, Ph.D.
Academic Editor
PLOS ONE

Dear Editor,

We have revised the manuscript according to Reviewers’ comments and journal requirements. We look forward to hearing from you. Thank you very much.

**Journal Requirements:**

Please review your reference list to ensure that it is complete and correct. If you have cited papers that have been retracted, please include the rationale for doing so in the manuscript text, or remove these references and replace them with relevant current references. Any changes to the reference list should be mentioned in the rebuttal letter that accompanies your revised manuscript. If you need to cite a retracted article, indicate the article’s retracted status in the References list and also include a citation and full reference for the retraction notice.

Response: We have checked the reference list and ensured that no retracted documents were cited in our manuscript. We have also revised the reference style according to the PLOS ONE's style requirements in the Revised manuscript.

[Note: HTML markup is below. Please do not edit.]

**Reviewers' comments:**

Reviewer's Responses to Questions

**Comments to the Author**
1. If the authors have adequately addressed your comments raised in a previous round of review and you feel that this manuscript is now acceptable for publication, you may indicate that here to bypass the “Comments to the Author” section, enter your conflict of interest statement in the “Confidential to Editor” section, and submit your "Accept" recommendation.

Reviewer #1: All comments have been addressed

Reviewer #2: All comments have been addressed

Reviewer #3: (No Response)

2. Is the manuscript technically sound, and do the data support the conclusions?

The manuscript must describe a technically sound piece of scientific research with data that supports the conclusions. Experiments must have been conducted rigorously, with appropriate controls, replication, and sample sizes. The conclusions must be drawn appropriately based on the data presented.

Reviewer #1: Yes

Reviewer #2: Yes

Reviewer #3: Yes

3. Has the statistical analysis been performed appropriately and rigorously?

Reviewer #1: Yes

Reviewer #2: Yes

Reviewer #3: Yes

4. Have the authors made all data underlying the findings in their manuscript fully available?

The [PLOS Data policy](http://www.plosone.org/static/policies.action#sharing) requires authors to make all data underlying the findings described in their manuscript fully available without restriction, with rare exception (please refer to the Data Availability Statement in the manuscript PDF file). The data should be provided as part of the manuscript or its supporting information, or deposited to a public repository. For example, in addition to summary statistics, the data points behind means, medians and variance measures should be available. If there are restrictions on publicly sharing data—e.g. participant privacy or use of data from a third party—those must be specified.

Reviewer #1: Yes

Reviewer #2: Yes

Reviewer #3: Yes

5. Is the manuscript presented in an intelligible fashion and written in standard English?

PLOS ONE does not copyedit accepted manuscripts, so the language in submitted articles must be clear, correct, and unambiguous. Any typographical or grammatical errors should be corrected at revision, so please note any specific errors here.

Reviewer #1: Yes

Reviewer #2: Yes

Reviewer #3: Yes

6. Review Comments to the Author

Please use the space provided to explain your answers to the questions above. You may also include additional comments for the author, including concerns about dual publication, research ethics, or publication ethics. (Please upload your review as an attachment if it exceeds 20,000 characters)

**Reviewer #1:** The revised manuscript entitled "The anti-tumor activity of tangeretin in esophageal squamous cell carcinoma by inhibiting GLI2-mediated transcription of GPNMB" is addressed all the concerns raised by reviewers.
The manuscript could be accepted after quality check especially the figures.

Response: We have improved the quality of the manuscript including the figures in the Revised manuscript.

**Reviewer #2:** The authors made necessary changes in the manuscript. I recommend accepting the manuscript. However, the following typos should be modified to provide a clear message to the readers.

1. Furthermore, Tan is the capacity of enhancing the sensitivity of the drugs and reducing the toxicity induced by chemotherapy

Response: We have changed the sentence to “Furthermore, Tan enhances the sensitivity of the chemotherapy drugs and reduces chemotherapy-induced toxicity” in the Revised manuscript.

2. A previous document proved the modulation of GPNMB in MMP9 in chronic obstructive pulmonary disease.

Response: We have changed the sentence to “A previous document proved that GPNMB can influence MMP9 expression in chronic obstructive pulmonary disease” in the Revised manuscript.

3. Therefore, while studying the anti-cancer mechanism of Tan, it is very important to study how overcomes these limitations for enhancing the therapeutic efficacy and reaching the clinical use

Response: We have changed the sentence to “Therefore, it is very important to study how these limitations are resolved for their clinical use.” in the Revised manuscript.

**Reviewer #3:** Remarks to the Author:
The article “The anti-tumor activity of tangeretin in esophageal squamous cell carcinoma by inhibiting GLI2-mediated transcription of GPNMB’’ is an interesting study. Minor revision is needed in recommending this manuscript for publication.
Minor issues:
The Quality of the images should be increased.
1. In Fig.1A, 1B, 1C, 1D need to be replaced with high resolution images. All the cell images 1B, 1C need scale bars as many of them lack scale bars into it. In fig. 1A and the graphs in Fig. 1B, 1C and 1D the font size should be increased for clear visibility.

Response: According to the comment, we have provided high resolution images and increased the font size in the Revised Figure 1.

2. In Fig.2 A and 2B all the fluorescent images needed scale bars into it. The font size of the graphs in these figures need to be increased.

Response: We have added scale bars for all the fluorescent images and increased the font size in the Revised Figure 2.

3. Fig. 3E needs to be changed with high resolution image.

Response: We have provided higher resolution images in the Revised Figure 3.

4. Fig. 4A, 4B, 4C font size need to be increased.

Response: We have increased the font size in the Revised Figure 4.

5. Fig. 6A, 6B need to be needs to be changed with high resolution images with increased font size.

Response: We have changed with high resolution images with increased font size in the Revised Figure 6A and 6B.

6. Font size of fig.7B needs to be increased.

Response: We have added the font size in the Revised Figure 7B.

7. Fig. 8C, 8D and 8E needs to be changed with high resolution images and scale bars must be added in each cell images.

Response: We have changed with high resolution images and added scale bars for all microscopy images in the Revised Figure 8.

8. Fig. 9A, 9B scale bars need to be added in each immunofluorescence cell images.

Response: We have added scale bars in each immunofluorescence cell images in the Revised Figure 9.

7. PLOS authors have the option to publish the peer review history of their article ([what does this mean?](https://journals.plos.org/plosone/s/editorial-and-peer-review-process#loc-peer-review-history)). If published, this will include your full peer review and any attached files.


If you choose “no”, your identity will remain anonymous but your review may still be made public.


**Do you want your identity to be public for this peer review?** For information about this choice, including consent withdrawal, please see our [Privacy Policy](https://www.plos.org/privacy-policy).

Reviewer #1: **Yes:** Jayaprakash Narayana Kolla

Reviewer #2: No

Reviewer #3: No

[NOTE: If reviewer comments were submitted as an attachment file, they will be attached to this email and accessible via the submission site. Please log into your account, locate the manuscript record, and check for the action link "View Attachments". If this link does not appear, there are no attachment files.]

While revising your submission, please upload your figure files to the Preflight Analysis and Conversion Engine (PACE) digital diagnostic tool, <https://pacev2.apexcovantage.com/>. PACE helps ensure that figures meet PLOS requirements. To use PACE, you must first register as a user. Registration is free. Then, login and navigate to the UPLOAD tab, where you will find detailed instructions on how to use the tool. If you encounter any issues or have any questions when using PACE, please email PLOS at [figures@plos.org](mailto:figures@plos.org). Please note that Supporting Information files do not need this step.

*In compliance with data protection regulations, you may request that we remove your personal registration details at any time.* [*(Remove my information/details)*](https://www.editorialmanager.com/pone/login.asp?a=r)*. Please contact the publication office if you have any questions.*
